# Mixing positive and negative valence: Affective-semantic integration of bivalent words

**DOI:** 10.1038/srep30718

**Published:** 2016-08-05

**Authors:** Michael Kuhlmann, Markus J. Hofmann, Benny B. Briesemeister, Arthur M. Jacobs

**Affiliations:** 1 Department of Education and Psychology, Free University Berlin, Habelschwerdter Allee 45, 14195 Berlin, Germany; 2 Department of Psychology, University Wuppertal, Max-Horkheimer-Str. 20, 42119 Wuppertal, Germany; 3 Center for Applied Neuroscience, Free University Berlin, Habelschwerdter Allee 45, 14195 Berlin, Germany; 4 Dahlem Institute for Neuroimaging of Emotion (D.I.N.E.), Free University Berlin, Habelschwerdter Allee 45, 14195 Berlin, Germany; 5 Center for Cognitive Neuroscience (CCNB), Free University Berlin, Habelschwerdter Allee 45, 14195 Berlin, Germany

## Abstract

Single words have affective and aesthetic properties that influence their processing. Here we investigated the processing of a special case of word stimuli that are extremely difficult to evaluate, bivalent noun-noun-compounds (NNCs), i.e. novel words that mix a positive and negative noun, e.g. ‘Bombensex’ (bomb-sex). In a functional magnetic resonance imaging (fMRI) experiment we compared their processing with easier-to-evaluate non-bivalent NNCs in a valence decision task (VDT). Bivalent NNCs produced longer reaction times and elicited greater activation in the left inferior frontal gyrus (LIFG) than non-bivalent words, especially in contrast to words of negative valence. We attribute this effect to a LIFG-grounded process of semantic integration that requires greater effort for processing converse information, supporting the notion of a valence representation based on associations in semantic networks.

Seek the positive, avoid the negative. A simple formula and useful basis for decision and action. The outcome of a positive/negative evaluation, a valence judgment, is therefore of utmost importance and integral to many theories of emotion. Most dimensional emotion theories starting with Wundt^1^,^2^,^3^,^4^,^5^ incorporate valence as the primal dimension together with a dimension for emotional activation or intensity (arousal). High valence words show systematic processing differences at the behavioral, experiential, and neural levels compared to neutral ones^6^,^7^ and there are also differences in the processing of positive and negative words^8^,^9^,^10^. The valence of the items that are used in the above-mentioned experiments is usually determined by large-sample rating studies. Databases like the Affective Norms for English Words (ANEW)^2^ or the Berlin Affective Word List - Reloaded (BAWL-R)^9^ are important tools for research on emotion and language^7^. On the other hand, such large-scale studies usually circumvent the issue of how the social construct ‘valence’ is neuronally represented and accessed: The evaluation process itself mainly is neglected and the ‘How’ of valence judgments still lacks proper (neuro)-cognitive modelling and explanation^7^.

Recent findings suggest that valence decisions may be supported by associations in semantic networks. For example, Hofmann and Jacobs^11^ found a correlation between the positive valence and the amount of associations of words. They showed that negative, but not positive valence induces false memory effects over and above those accounted for by the amount of associated words in the stimulus set. In a similar vein, Westbury *et al*.^12^ predicted the valence ratings of words by their association to a selected set of emotion labels taken from extant emotion theories. In general, semantic network models assume that the building and consolidation of semantic associations follows a Hebbian learning mechanism^13^. When two words systematically appear spatio-temporally close together their association is strengthened. These associations are estimated in a reversed approach by counting the events of co-occurrence within large text corpora, and correcting them for individual word frequencies. While computation of semantic associations can straightforwardly explain effects for words with a clear positive and negative valence, words also can have a mixed affective structure and thus the question arises how a mixed valence is represented and processed^7^.

When rated separately for positivity and negativity, some words score high on both scales^14^. Thus, like other non-verbal stimuli^15^ words can be perceived as being positive and negative at the same time. Here, we would like to propose that the evaluation of such bivalent words engages an affective-semantic integration process. In Panksepp’s hierarchical emotion theory^16^,^17^ valence is considered a cognitive emotion component of the tertiary process level that is supported by neocortical areas. Therefore, the evaluation of word valence engages activation in cortical areas such as the inferior frontal gyri^18^. In a recent test of Panksepp’s theory using both EEG and fMRI with human subjects, Briesemeister *et al*.^19^,^20^ showed that positive valence in comparison to happiness (as a discrete emotion attributed to the primary process level) recruits neocortical activity. These authors suggested a semantic foundation of this affective dimension. Following up on this work, Jacobs *et al*.^7^ recently proposed that valence and arousal are semantic superfeatures that result from a yet unknown integration of both experiential and distributional data, as assumed by the semantics theory of Andrews *et al*.^21^.

## The present study

Inspired by the above mentioned research, we hypothesized that affective-semantic integration recruits the left Inferior Frontal Gyrus (LIFG), which is suggested to be a key region for integration processes, e.g. in the Memory, Unification, Control framework (MUC)^22^. Neurocognitive evidence for this assumption comes from a study by Forgács *et al*.^23^, who reported that newly combined noun-noun compounds (NNCs) engage semantic integration activity in the LIFG. More recently, Hofmann and Jacobs^11^ used theoretical word association strengths as simulated by the Associative Read-Out Model (AROM)^24^ to predict LIFG activation generated by the NNC stimuli of Forgács *et al*.^23^.They found that strong long-term associations between the constituent words of an NNC effectively inhibited the integrative function of the LIFG.

A particularly interesting group of words to probe affective semantic integration processes are affectively bivalent words in the form of novel (German) NNCs which combine two positive or negative constituents, a positive with a negative constituent, or vice versa^7^. In contrast to most English NNCs, German NNCs are assembled by writing two or more words together without a dividing space. With regard to studying integration processes, this is an advantage, because undivided compounds are processed faster than those divided by a space^25^. In German like in all Germanic languages compounds are left-branching and the rightmost constituent is the head which defines the meaning, while all other constituents are modifiers constraining the meaning^26^. In German compounds the use of interfixes, inserted letters that serve as a linking element, is fairly common. The most frequent is the letter ‘s’. This interfix also exists in English (e.g., salesman) and is a possessive inflection. Compounds contain the information of a minimal sentence of the structure ‘modifier has head’ (e.g., Fingerabdruck - fingerprint) or ‘head is for modifier’ (e. g., Regenmantel - raincoat). They are an important mechanism for the creation of new words (e.g., internet). The meaning of a compound may result from the combination of the meaning of the constituents (e.g., blueberry) thus being ‘transparent’. In the case of ‘opaque’ compounds the meaning of a constituent is unassociated with the meaning of the compound (e.g., ‘straw’ in strawberry). Compounds and similar word pairs thus engage semantic integration processes which is especially true for new compounds^23^,^27^,^28^.

When a compound made it into common usage, however, there is less need to derive the meaning gestalt by integrating the meanings of its constituents. The meaning of the compound is then hypothesized to be directly represented in semantic memory, much like a ‘dead’ metaphor. In contrast, the meaning of novel compounds can only be constructed by integrating the meaning of the constituents. Structure mapping theory^29^ suggests that the structure of one meaning must be mapped onto the other, and that comprehension is achieved by comparing the relations of the attributes of the words. Sopory^28^ suggested that valence can be considered such an attribute. Thus, because bivalent compounds consist of differential attributes, it should be more difficult to integrate their seemingly irreconcilable affective meanings.

Bivalent NNCs of the type we used in this study were already tested in behavioural pilot experiments partly reported in Jacobs *et al*.^7^. In a valence decision task (VDT) the responses to bivalent NNCs (i.e., positive-negative or vice versa) showed a negativity bias: far more often they were categorized as negative than as positive. Reaction times (RTs) were extremely slow and expectably slower than for control NNCs with equal valence constituents (e.g., positive-positive). These non-bivalent NNCs were usually evaluated in accordance with the valence of the constituents.

Here, we hypothesized that non-bivalent NNCs would not engage semantic integration processes, or to a much lesser degree, than the bivalent ones. To deal with the contradicting valence of the constituents, the latter should fully engage the LIFG, as can be expected from Hagoort’s^22^ MUC theory.

## Methods and Materials

### Participants

The 24 participants (7 male; aged 19–29; mean 23.38) who took part in our fMRI study were right handed, had normal or corrected-to-normal vision and were native speakers of German. They were recruited at the Free University Berlin and gave written informed consent. They either received course credit or were paid for participation. The study was carried out in accordance with the approved guidelines, and was also approved by the ethic committee of the Free University Berlin.

### Stimuli

A set of 120 NNCs (see Supplementary Table S1) was generated by randomly pairing nouns that met our requirements for general positive or negative valence taken from the BAWL-R^9^. The mean valence ratings are given in Table 1. Valence ranged from −3 to −1.3 for negative, and from 1.3 to 3 for positive words (total scale is 7 points ranging from −3 for very negative through 0 for neutral to + 3 for very positive) and the absolute value was larger than 1.5 times the individual standard deviation. Word length was restricted to 5 to 8 letters per word, resulting in compounds with 10 to 16 letters length.

The set was subdivided into four conditions defined by the valence of the constituents (positive-positive, negative-negative, positive-negative, negative-positive). The two conditions with incongruent constituents formed the bivalent compounds.

All compounds were manually inspected and where appropriate, a binding letter was inserted in accordance with German morphosyntactic rules of compound construction. We cross-checked each compound with the data base of a standard German dictionary (Duden) to ensure that they were actually novel. In a pilot rating study (n = 36) we checked the comprehensibility and imageability of the new compounds on 7-point Likert scales (0 = uncomprehensible/non-imageable to 7 = comprehensible/imageable). Every single compound had an average comprehensibility score of above 3 (m = 4.33, sd = 0.58) indicating no uncomprehensible items. Comprehensibility and imageability of compounds did not differ significantly across conditions (ANOVAs, all *F*(3, 116) < 1; ns). In addition, we checked that imageability, word frequency (CELEX)^30^, letter, and syllable count of constituents did not significantly differ across conditions (ANOVAs, all *F*(3, 116) < 1; ns).

### Procedure

The participants first received general information about the study and magnetic resonance imaging. They were then placed in the scanner by trained personal. The participant’s right hand was placed on the response box and with their left hand they had the possibility to press an emergency button at any time to signal abortion of the measurement. The screen, on which the stimuli were presented, was visible via a mirror system.

In a VDT the participants had to categorize each presented item as either positive or negative. Responses were given via the right hand’s index and middle finger on the buttons of a response box. The response mapping was balanced across participants. The participants were instructed to respond within the time window of presentation (3000 ms).

The instructions of the experiment were presented in written form on the screen. Each task began with 10 practice trials. Before and after the practice block the participants were asked if any questions remained open.

The design was event-related and the items were presented in Optseq2^31^ calculated random order in two 60-trial blocks. Between the two blocks the participants could take a break. A trial began with the presentation of a fixation cross for between 1500 ms and 6500 ms, jittered in steps of 500 ms, in the centre of the screen. These durations were calculated with Optseq2 to ensure a maximal signal-to-noise ratio. The fixation cross was replaced by the item, which was presented for 3000 ms. All blocks were set to a fixed length of 200 volumes. Because of inhomogeneous timings due to the jittered intervals, after the last trial of a block a fix cross was presented till the end of the block.

### Image acquisition

Scanning was done with a Siemens Tim Trio 3 tesla scanner (Siemens, Erlangen, Germany) with a standard 32 channel head coil at the Dahlem Institute for Neuroimaging of Emotion (D.I.N.E.) in Berlin. The parameters of the standard EPI sequence were TR = 2000 ms, TE = 30 ms, flip angle = 70°, 37 axial slices oriented along the AC-PC plane, interleaved from bottom to top, 3 mm slices, no gap, 3 mm × 3 mm, in-plane. A high resolution T1-weighted image for anatomical localization was collected and fMRI localized shimming reduced susceptibility artefacts.

### Behavioural analysis

RT data from the VDT were analysed with a mixed fixed and random effects model using the Statistical software JMP 11 (SAS Institute Inc.). The type of compound (positive-positive, negative-negative, positive-negative, negative-negative) was modelled as a fixed effect, participants, and items nested within conditions were modelled as random effects. We analysed with χ^2^-Tests if there were contingencies between the participants’ responses and the valence of the constituents.

### fMRI analysis

All FMRI data analyses were conducted with the SPM8 (http://www.fil.ion.ucl.ac.uk/, downloaded: 11-14-2013) toolbox for Matlab (Mathworks, Inc.). Images were first slice time corrected, realigned to the first image of the volume and simultaneously unwarped. All interpolations were done with 4th degree B-spline. Images were coregistered with the image of the anatomical scan. A group anatomical template was created from the segmented white and grey mater images of the anatomical images of all participants using DARTEL (Diffeomorphic Anatomical Registration using Exponentiated Lie algebra)^32^. The template was used to normalize functional images to MNI-space (Montreal Neurological Institute). Thereby we achieved a homogeneous structural organization of brain anatomy for all participants.

Trials without response were excluded from the analysis. For each subject we specified and estimated a general linear model (GLM). Movement regressors from preprocessing were included in the computation and we build T-contrasts for each of the four factor combinations: positive-positive, negative-negative, positive-negative, negative-positive. Based on these T-contrasts a single GLM was computed for the whole sample. We calculated main effect T-contrasts for modifier and head of the compounds. For the interaction of modifier and head we calculated a contrast with all of the factor combinations and another contrasts that compares congruent (positive-positive and negative-negative) vs. incongruent (positive-negative and negative-positive) combinations.

## Results

### Behavioral data

**Figure 1:**
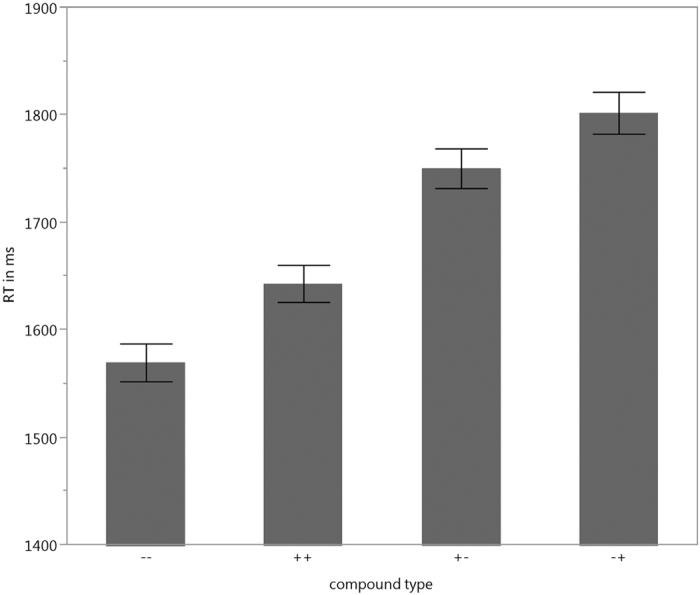
Reaction Times of the VDT (error bars represent standard error).

The linear mixed effects model showed a main effect of condition, *F*(3,115.9) = 13.4; *p* < 0.001. The effect was driven by faster responses to congruent conditions than to incongruent conditions (Fig. 1). Pair-wise comparisons with Bonferroni correction revealed a significant difference for the negative-negative-condition compared to both incongruent conditions, i.e. positive-negative, *t*(115.86) = 4.4; *p* < 0.001, and negative-positive compounds, *t*(115.86) = 5.74; *p* < 0.001. The other congruent condition, positive-positive, only differed significantly from the incongruent condition negative-positive, *t*(115.86) = 4.02; *p* < 0.001. The comparison with the other bivalent condition only approached significance, *t*(115.86) = 2.68; *p* = 0.051. The congruent conditions, positive-positive and negative-negative, did not differ significantly from each other, *t*(115.86) = 1.73; *p* < 0.52; neither did the incongruent conditions, positive-negative and negative-positive, *t*(115.86) = 1,34; *p* = 11.058. Responses to non-bivalent compounds were generally related to the valence of the constituents. For the bivalent compounds there was a strong tendency towards a negative response. In other words, when one of the constituents in the compound was negative the response was also likely to be negative, which could be confirmed with a significant χ^2^-Test, χ^2^ (1, *N* = 1866) = 546,59; *p* < .0001.

### Neuroimaging results

**Figure 2:**
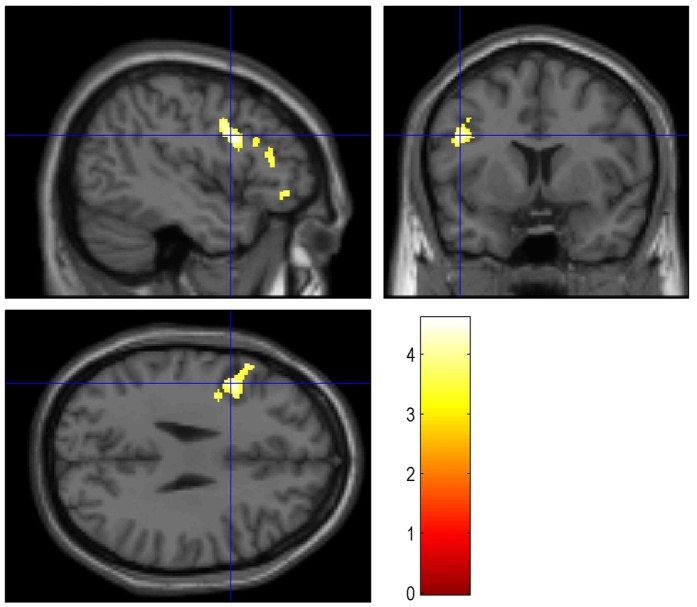
Activation in the LIFG from contrast: Bivalent > Non-bivalent (the cross-hair marks the voxel of maximal activation, the colour-bar represents t-values).

**Figure 3:**
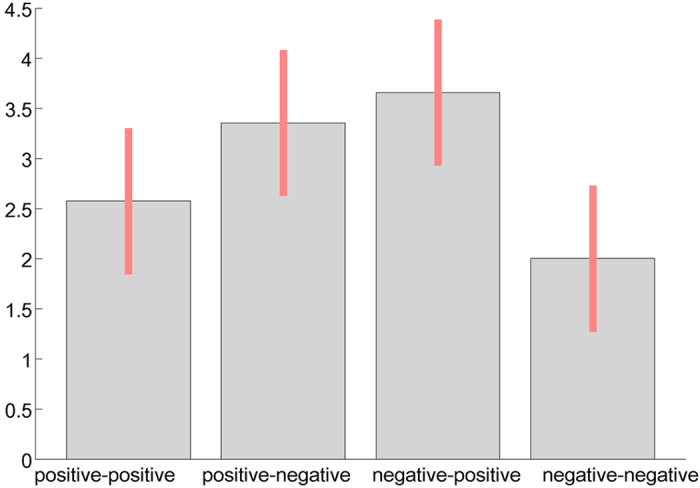
Percent signal change of BOLD-signal at LIFG peak voxel (−45, 8, 28) with red bars representing standard error.

In line with our hypothesis that bivalent NNCs engage semantic integration processes we observed strong activation in the LIFG. All reported effects were significant at a cluster level of *p* < 0.05 corrected for family-wise error and surpassed a cluster size threshold of 10 voxels. There were no main effects for the valence of the compounds’ modifier or head. The contrast bivalent vs. non-bivalent compounds (Fig. 2) revealed a single large cluster of significant activity differences. This cluster was mainly located in the LIFG, and peaked there in activity (Table 2), but also partially extended into the left precentral gyrus (LPCG). An F-contrast of all factor combinations showed a gradual increase of BOLD-signal at the peak voxel of the LIFG cluster (Fig. 3). The increase in the signal was in the following order: negative-negative, positive-positive, positive-negative, and negative-positive compounds.

## Discussion

When the valence of a novel compound has to be evaluated, the incongruent valences of its constituents can make this task difficult, as can be seen by longer RTs for bivalent NNCs^7^. In the present study we not only replicated this finding, but we also hypothesized that this effect results from the effort required for semantic integration. Hagoort^22^ suggested that the LIFG integrates different types of information and among others presented supportive neuroimaging findings from a study with semantic violations^33^. In a similar vein the constituents of bivalent NNCs are semantically more dissociated and we therefore expected greater LIFG activation to bivalent than to non-bivalent compounds. The present results confirmed this hypothesis. This result may be explained by structure mapping theory^29^ suggesting that comprehension is achieved by comparing relations among attributes. For bivalent NNCs that comparison will result in fewer affective similarities than for non-bivalent NNCs, whose constituents share the same valence. Therefore, meaning making is more difficult for bivalent NNCs. It is questionable, however, whether the process of affectively toned meaning making is hosted more generally in bilateral inferior frontal regions. This is suggested by a PET study showing activity in the right inferior frontal gyrus to occur only if an explicit affective judgment of bivalent stimuli was required^34^.

A more detailed inspection of our BOLD-signal change at the peak voxel of LIFG activity revealed that the positive-positive compounds also elicited considerable LIFG activity in a magnitude between the negative-negative and the bivalent compounds. Again, this conforms to our behavioural data that show an RT advantage for negative-negative NNCs vs. positive-positive NNCs. Increased LIFG activity for positive-positive NNCs as compared to negative-negative ones may be explained by the number of associations (NA): Positive words usually have a higher number of associations than negative words^11^. Therefore, they may be more prone to elicit semantic integration. However, the influence of general NA on the process of semantic integration seems to be smaller than the influence from the semantic dissociation of the constituents. Bivalent compounds elicited the strongest LIFG activity and longest RTs, but they have a negative constituent and therefore introduce less average NA than the positive non-bivalent NNCs. The NA account could also be tentatively framed within structure mapping theory^29^, because a greater NA value may trigger more comparison processes until all similarities are found and semantic integration is reached. The constituents of negative-negative NNCs, in contrast, have fewer associations with many similarities, which should render meaning making easiest for them.

It is worth noting that in order to evaluate the valence of a compound, the meaning of both target words must be integrated. This contrasts with semantic priming, in which the decision concerns only one target word^35^. Nevertheless, we think that also in priming the meaning of the prime and the target are incidentally integrated, thus eliciting IFG effects^36^. However, in contrast to semantic priming, compound evaluation requires the semantic integration process to be relatively finished before a response can be given. This time consuming processing explains why the present RTs are twice as large as typical RTs in a semantic priming experiment^35^. Since no evidence was found suggesting that one of the constituents, ‘modifier’ or ‘head’, has a specific influence on the evaluation of NNCs, we think it safe to say that the evaluative decision in our experiment is based on the integrated information.

In sum, our findings further support the notion that the valence of a word is also derived from distributional data^7^. In our experiment we used novel NNCs, which precludes direct affective experience with that stimulus as a source of information. Hence the evaluation is solely grounded in associated affective information derived from the constituents of the NNCs and integrated in accordance with theories of metaphoric meaning making^28^,^29^.

### Limitations and outlook

Our study was conducted with novel German NNCs. We think that the German language is very well suited to study effects of semantic integration, because of its strong productivity concerning compounds. Although they are common to most languages, compounds are especially abundant in German, and have a simple orthography, as they are all left branching and usually written as one word without interrupting spaces. We expect that LIFG-based semantic integration of novel compounds takes place in other languages too, but if the rules for compounds are more diverse than in German, this may affect their processing. Some languages, like Italian for example, have right and left branching compounds with differences in their lexical processing^37^. In English there is a more diverse compound orthography, with closed compounds (e.g., girlfriend), hyphened compounds (e.g., “credit-rating”), and open compounds (e.g., “tennis racket”), and again lexical processing and semantic integration might be different^38^. Therefore, an intriguing sequel might ask how such nuances in form, or how a novel compound becoming lexicalized, affect semantic integration and meaning making. Finally, future research may tackle the question whether affective incongruence produces a more vivid memory of the compound, e.g. in recognition memory tasks known to produce false alarms^24^.

## Additional Information

**How to cite this article**: Kuhlmann, M. *et al*. Mixing positive and negative valence: Affective-semantic integration of bivalent words. *Sci. Rep.* **6**, 30718; doi: 10.1038/srep30718 (2016).

## Supplementary Material

Supplementary Information

## Figures and Tables

**Table 1:** Means and Standard Errors for lexical and semantic Variables for the constituents.

Variable	Non-bivalent Compounds				Bivalent Compounds			
(m) = modifier (h) = head	Positive-Positive		Negative-Negative		Positive-Negative		Negative-Positive	
	M	SE	M	SE	M	SE	M	SE
Valence (m)	1.98	0.36	−1.89	0.27	1.79	0.29	−1.88	0.33
Valence (h)	1.86	0.31	−1.84	0.34	−1.89	0.3	1.73	0.27
Imageability (m)	4.42	1.33	4.08	1.06	4.06	1.25	4.31	1.1
Imageability (h)	4.24	1.36	4.20	0.95	4.22	1.21	4.55	1.19
Word frequency (m)	17.76	22	11.24	20.02	15.31	19.75	11.27	12.97
Word frequency(h)	13.82	18.59	8.09	10.15	9.11	14.08	11.49	16.71
#Letters (m)	6.87	1.04	6.47	1.14	6.4	1.04	6.63	1.07
#Letters (h)	6.4	1.19	6.7	1.09	6.8	1.03	6.4	1.07
#Syllables (m)	2.1	0.4	2.1	0.4	2.3	0.53	2.1	0.66
#Syllables (h)	2.17	0.53	2.13	0.63	2.03	0.49	2.13	0.43

**Table 2:** Brain regions showing significantly greater BOLD signal (*p* FWE-corr < 0.001).

Condition	Region	#Voxels	BA	x	y	z	t-value
Bivalent > Non-bivalent	L inferior frontal gyrus	1213	6/44	−45	8	28	4.60
	L precentral gyrus	269	6				
