# Supplementary material for: Mixing positive and negative valence: Affective-semantic integration of bivalent words
Source: Sci Rep. 2016 Aug 5;6:30718. doi: 10.1038/srep30718 (PMC4974501; doi:10.1038/srep30718)
Supplement: Supplementary Information [file srep30718-s1.pdf]

Mixing positive and negative valence:
Affective-semantic integration of bivalent words

Michael Kuhlmann [a*], Markus J. Hofmann [b], Benny B. Briesemeister [c], Arthur M. Jacobs [a, d, e]

[a] Department of Education and Psychology, Free University Berlin, Habelschwerdter Allee 45, 14195 Berlin, Germany, michael.kuhlmann@fu-berlin.de (corresponding author)

[b] Department of Psychology, University Wuppertal, Max-Horkheimer-Str. 20 42119 Wuppertal, Germany, mhofmann@uni-wuppertal.de

[c] Center for Applied Neuroscience, Free University Berlin, Habelschwerdter Allee 45, 14195 Berlin, Germany, benny.briesemeister@fu-berlin.de

[d] Dahlem Institute for Neuroimaging of Emotion (D.I.N.E.), Free University Berlin, Habelschwerdter Allee 45, 14195 Berlin, Germany, ajacobs@zedat.fu-berlin.de

[e] Center for Cognitive Neuroscience (CCNB), Free University Berlin, Habelschwerdter Allee 45, 14195 Berlin, Germany, ajacobs@zedat.fu-berlin.de

Table S1: List of Items

Positive-Positive	Negative-Negative	Positive-Negative	Negative-Positive
Aussichtstoleranz	Abszessschock	Anmutfeigling	Absagearoma
Duschvenus	Ärgerhenker	Anrufinzest	Abschaumknospe
Einfallvorsorge	Arrestschuft	Aromaunwesen	Abstiegsgarten
Einklangsschlaf	Blödheitskargheit	Ausflugunrecht	Armutswunsch
Erholungsjubel	Bombenmutation	Bildungsgalgen	Bomberumsicht
Erotikengel	Bunkersadismus	Diplomübelkeit	Choleravilla
Fantasiediplom	Einsturzmakel	Ekstasetrübsinn	Drecksausdauer
Freiheitsgenie	Geißelsintflut	Friedekerker	Feiglingsgeschenk
Gartenaussicht	Killerpickel	Frühlingszweifel	Fluchjugend
Genusseinfall	Kummernotfall	Helferschleim	Furchtfrühling
Geschenktugend	Lepraelend	Jugendfreitod	Gesindeltulpe
Gewinngenießer	Lügnertorpedo	Karrierepeiniger	Grippeklugheit
Heilungsquelle	Makelabstieg	Kissenlepra	Halunkenekstase
Hobbyhoffnung	Milizseuche	Kontaktmeineid	Henkerhelfer
Hoffnungsnatur	Monstrumverfall	Konzertpranger	Ignoranzkakao
Klugheitsumarmung	Neurosearmut	Massagesaboteur	Jaucheheimat
Künstlerliebreiz	Pickelhorror	Medaillenbiest	Knastluxus
Lorbeererotik	Plagenmoder	Medizinkomplott	Konfliktoptimist
Orgasmusheilung	Prellungsallergie	Naturmonstrum	Marschtraube
Partnerretter	Prügelzerrung	Optimistkonflikt	Marterpurpur
Purpurflirt	Racheverlust	Romanzenabschied	Migränehobby
Respektklarheit	Saboteurfluch	Scherzführer	Mottenkonzert
Rettungsbargeld	Schockrechnung	Sesselgeißel	Prangerkamin
Rubinküken	Sintflutmigräne	Siestavorwurf	Rechnungsmelodie
Saphirseide	Übelkeitsleichnam	Sprachenzerfall	Schlägersieger
Schutzsiesta	Unfriedebastard	Toleranzfieber	Torpedobalkon
Siegersprache	Verfallskummer	Tugendrheuma	Verrätertriumph
Trefferbonbon	Verlustgesindel	Welpenkollaps	Verratsvorspiel
Vorspielmedizin	Vorwurfsgrippe	Willebombe	Zerrungsrespekt
Waffelanmut	Zerfallstrennung	Wonnedreck	Zweifellorbeer
